# Supplementary material for: Optimising older adults’ home spaces to enhance their physical activity level: an exploratory qualitative study protocol
Source: BMJ Open. 2023 Feb 16;13(2):e066940. doi: 10.1136/bmjopen-2022-066940 (PMC9936290; doi:10.1136/bmjopen-2022-066940)
Supplement: Supplementary data [file bmjopen-2022-066940supp001.pdf]

Project Title:
Optimizing older adults home spaces to enhance their physical activity level: An exploratory qualitative study protocol

Demographic Information

1.GENDER	
2. AGE	
3.Your highest education level	1. No formal education<br>2. Less than primary school<br>3. Primary school completed<br>4. Secondary/High school completed<br>5. College/University completed<br>6. Post graduate degree
4. Your occupation	1. Government employee<br>2. Non-government employee<br>3. Self-employed<br>4. Non-paid<br>5. Retired<br>6. Unemployed (able to work)<br>7. Unemployed (unable to work)<br>8. Other, specify:
5. How many people live in your house now (**not** counting yourself)	____________ people<br>[insert number]
6. Number of families in the house:	1. One (i.e., husband and children)<br>2. Two or more (e.g., in-laws, brother-in-law and his family, other relatives like aunts, uncles)
7. What is your nationality?	1. Pakistani<br>2. Indian<br>3. Chinese<br>4. Bangladeshi<br>5. African<br>6. White<br>7. Other___________________________________
8. What is your current marital status?	1. Married<br>2. Living as couple (living together, but not legally married)<br>3. Divorced or separated<br>4. Single, never married<br>5. Widowed<br>6. Other (Specify):<br>____
9. Did you have any significant medical history? (check all that apply)	1. Heart attack<br>2. Heart surgery or cardiac angiogram or insertion of stents<br>3. Angina<br>4. Pacemaker or implantable cardiac defibrillator<br>5. Rhythm disturbance (irregular or fast heart rate)<br>6. Heart valve disease

	7. Heart failure<br>8. Heart transplantation<br>9. Congenital heart disease<br>10.High blood pressure<br>11. Diabetes<br>12. Renal disease<br>13. Asthma or any other lung condition<br>14. Any musculoskeletal (joint or muscle) problems that may limit your physical activity<br>15. Other _______________
10.Have you taken any prescription medications	1. Yes<br>2. No<br><br>If yes, please list the medications you have been taking:<br><br>________________________

Indoors vs Outdoors

Some people describe themselves are being 'outdoorsy' or “indoorsy’ people. How would you describe yourself? *Prompt: Would you say you are more indoorsy or outdoorsy? Do you like being active like walking in your spare time? Or doing things like watching TV, being on the computer or reading?*

Why do you say that? What makes you choose to do something active like walking in the garden vs watching TV ?

1. Thinking about your home. What are your favorite features of the house and garden ? What do you most like and why?
2. What do you most dislike and why?
3. What kind of activities do you perform at home?
4. Can you tell me the number of days in a usual week when you were physically active for at least 150-300 minutes or 2.5 hour to 5 hours?
5. What are your ways of being physically active at home?
6. What obstacles stand in the way of physical activity in your home setting?
7. Do you have access to mobile technology like laptops, smart phone, handheld electronic game etc?
8. Where do you usually use these at home? Where do you usually access the internet at home?
9. Can you tell me the time spent watching TV, playing video and computer games, and using the internet for leisure, on a typical week and weekend day?
10. Do you have any rules around media use we haven’t already spoken about? Tell me about these. Prompt: When? Where? How long? Do these apply to the whole family?
11. Why did you choose this home for your family/yourself to live in? What things did you consider?
12. Why is your home organized in the way that it is at present?
13. Have you changed anything about your home over the past 5 years (e.g., technical equipment or device and the reason of these changes in home?
14. If you could change anything about your home, what would it be? Why would you do this?
15. What if you could redesign your house to do things like rearrange the rooms or make areas bigger or smaller? What would you do?
16. What if you had to make changes to the house and garden to make it more active? What would you do?
17. What if you had to make changes to the house & garden to get away from screens and spend less time on the couch? What would you do?
18. Do you have any physical activity equipment (treadmill, exercise bike, weights/resistance band) within your home?
19. Do you have exercise programming app (smartphone exercise apps, exergames, work out videos, virtual personal trainer, virtual workout groups)
20. Are there any parks nearby your home and would you prefer to go there?

21. What do you think that pandemic has restricted your physical activity level within your home surroundings? (Prompt: Is there any change in daily routine)
22. What do you think that currently your home physical environment (example space within home) promotes your health and well-being? (Prompt why? What are the factors?)
23. What are your suggestions and recommendations for older people in terms of optimizing their home space to be more physically active?
