# Supplementary material for: Optimising older adults’ home spaces to enhance their physical activity level: an exploratory qualitative study protocol
Source: BMJ Open. 2023 Feb 16;13(2):e066940. doi: 10.1136/bmjopen-2022-066940 (PMC9936290; doi:10.1136/bmjopen-2022-066940)
Supplement: Author's manuscript [file bmjopen-2022-066940.draft_revisions.pdf]

BMJ Open is committed to open peer review. As part of this commitment we make the peer review history of every article we publish publicly available.

When an article is published we post the peer reviewers' comments and the authors' responses online. We also post the versions of the paper that were used during peer review. These are the versions that the peer review comments apply to.

The versions of the paper that follow are the versions that were submitted during the peer review process. They are not the versions of record or the final published versions. They should not be cited or distributed as the published version of this manuscript.

BMJ Open is an open access journal and the full, final, typeset and author-corrected version of record of the manuscript is available on our site with no access controls, subscription charges or pay-per-view fees (http://bmjopen.bmj.com).

If you have any questions on BMJ Open's open peer review process please email info.bmjopen@bmj.com

BMJ Open

Optimizing older adults home spaces to enhance their physical activity level: An exploratory qualitative study protocol

Journal:	*BMJ Open*
Manuscript ID	bmjopen-2022-066940
Article Type:	Protocol
Date Submitted by the Author:	28-Jul-2022

Complete List of Authors:	Meghani, Naureen; Swansea University College of Engineering - Bay Campus; Swansea University College of Engineering,<br>Hudson, Joanne; Swansea University College of Engineering - Bay Campus, Applied Sports Technology, Exercise and Medicine (A-STEM) Research Centre<br>Stratton, Gareth; Swansea University College of Engineering - Bay Campus, College of Engineering<br>Mullins, Jane; Swansea University School of Engineering

Keywords:	QUALITATIVE RESEARCH, MENTAL HEALTH, Old age psychiatry < PSYCHIATRY

SCHOLARONE™
Manuscripts

TITLE PAGE

Study Title: Optimizing older adults home spaces to enhance their physical activity level: An exploratory qualitative study protocol

Naureen Akber Ali Meghani[1], Joanne Hudson[2], Gareth Stratton[3], Jane Mullins[4]

Details of the Authors:

[1]Naureen Akber Ali Meghani, PhD Student, Applied Sports Technology, Exercise and Medicine (A-STEM) Research Centre, Swansea University, Bay Campus, Swansea, United Kingdom, 2132644@ swansea.ac.uk . +07762330193

[2]Joanne Hudson, Professor of Exercise and Sport Psychology, Applied Sports Technology, Exercise and Medicine (A-STEM) Research Centre, Swansea University, Bay Campus, Swansea, United Kingdom, joanne.hudson@swansea.ac.uk 01792 513162

[3] Gareth Stratton, Chair in Paediatric Exercise Science, College of Engineering, Swansea University, Bay Campus, Swansea, United Kingdom, g.stratton@swansea.ac.uk 01792 516544

[4] Jane Mullins, Researcher, College of Engineering, Swansea University, Bay Campus, Swansea, United Kingdom, j.mullins@swansea.ac.uk 07954690170

Corresponding Author Detail:
Naureen Akber Ali Meghani
PhD Student,
Swansea University,
Bay Campus (SA1 8EP), Swansea, United Kingdom,
Cell Phone: +07762330193
Email: 2132644@swansea.ac.uk .

Keywords: Qualitative study, older adults, physical activity level, home spaces, exploratory study

Word Count: 3033

For peer review only - http://bmjopen.bmj.com/site/about/guidelines.xhtml

Abstract

Introduction:

Housing is a major contributing factor to health, and better housing condition has been linked to improved general and psychological health. There has also been strong evidence that the physical environment within the home setting substantially impacts sedentary behaviour and physical activity in children. However, there is a lack of research that has investigated the physical environment within the home setting in the context of older adults' physical activity levels and sedentary behaviour. Given that with increasing age, older adults spend large proportions of their time in their homes it is important to optimize older adults' home settings to support healthy ageing. Therefore, the current study aims to explore older adults' perceptions around optimizing their physical environment within their home space to support physical activity and subsequently facilitate healthy ageing.

Methods and analysis:

In this formative research, a qualitative exploratory research design using in-depth interviews (IDIs) and a purposive sampling approach will be employed. In-depth interviews will be conducted to collect data from study participants. Older adults from diverse community organizations in Swansea, Bridgend, and Neath Port Talbot will formally request approval to recruit via its contacts for this formative research. The study data will be analyzed thematically using NVivo V.12 Plus software.

Ethics and dissemination:

Ethical approval for this study has been obtained from the College of Engineering Research Ethics Committee (NM_31-03-22), Swansea University. The findings of the study will be disseminated to the scientific community and to the study participants. The results will enable

For peer review only

us to explore the perceptions and attitudes of older adults towards physical activity within their home environment.

Key words: Qualitative study, older adults, physical activity level, home spaces, exploratory study

Strengths and limitations of this study:

- This exploratory qualitative study will provide an in-depth understanding of how older adults' homes spaces support or prevent physical activity and sedentary behaviour which in turn will impact on health.
- The findings of this study will guide the development of context-specific interventions to address the challenges of optimizing physical activity within older adults' home spaces.
- This study will also guide the formulation of approaches and strategies to improve physical activity and decrease sedentary behaviour
- In addition, the study will contribute to social care policy that adopts a preventive healthcare model to maintain older adults' wellbeing by enabling them to be independent in their home surroundings.
- This will create awareness about older adults' engagement within their home setting that guide us to integrate novel insights about optimizing their home space.
- One of the major limitations of the study might be that some semi-structured interviews will be conducted online via Zoom or WhatsApp depending on the older adult's preference which might serve as a barrier to recording non-verbal cues during interviews.

For peer review only - http://bmjopen.bmj.com/site/about/guidelines.xhtml

Background:

Physical activity (PA) is a key factor of healthy ageing; however, many older adults are not sufficiently physically active and have sedentary lifestyles which leads to detrimental health consequences, and even places financial and social constraints on themselves and society (1). This is a global concern, with longevity increasing around the world, it is estimated that proportion of older adults in the global population is expected to nearly double from roughly 12% to 22% between 2015 and 2050, translating to an increase from 900 million to 2 billion people over the age of 60 (2). However, it is important to note that the aforementioned increase in life expectancy may not necessarily imply an increase in healthy life expectancy, as older people can face a variety of health issues (3). These include cognitive function decline, reduced functional movement, impaired mental health and chronic conditions such as diabetes, high blood pressure, and cardiovascular disease (3). These health issues can have an impact on an older person's quality of life (4), and increase healthcare costs and societal burden (e.g., medical care expenses; (5). According to estimates, physical inactivity contributes to the worldwide burden of noncommunicable diseases (NCDs), with health-care systems costs attributed to inactivity estimated at INT$53.8 billion globally, and further costs linked with INT$13.7 billion in productivity losses (6, 7). Reducing the mortality rates attributable to NCD calls for considerable action and equitable access to effective and good quality preventative and curative care, including behaviour modification such as increasing PA (8). In that regard, engagement in activity (as part of leisure, sport, work, and transportation) plays a significant role in successful ageing (9-11). Thus, developing strategies that are tailored to meet the needs of different subgroups (e.g., older adults) and that account for cultural contextual influences on behaviours related to, and attitudes towards, healthy ageing is paramount. Doing so will enable us to identify strategies to combat the effects of noncommunicable diseases, improve and preserve functional capacity, and promote physical and mental health among older persons to

For peer review only - http://bmjopen.bmj.com/site/about/guidelines.xhtml

support independence and improve quality of life (11, 12). Therefore, there is an urgent need to increase older adults' PA levels and decrease their sedentary behaviour.

The World Health Organization has placed a particular emphasis on the promotion of PA to support healthy ageing (13). The Global Action Plan on Physical Activity 2018-2030 identifies the strategic goal of increasing PA opportunities to engage inactive populations such as older adults (14). Of concern, studies have shown that some older adults perceive physical activity to be only beneficial to the younger population (15); however, there is compelling evidence that the requirement for physical activity does not end in later life (16). Doing so has numerous benefits, including a positive influence on the ability to perform the daily living activities (17, 18), and findings from longitudinal studies demonstrate that engaging in regular physical activity can improve cognitive ability among older adults (16). Findings from systematic review and meta-analysis showed that physically active older adults exhibited much lower arterial stiffness as compared to their sedentary peers (standardized mean difference: -1.017 ± 0.340, 95% CI: -1.684 to -0.350, p = 0.003). Further, there is a drastic decline in the risk of cardiovascular disease mortality from 25% to 40% that accompanies low and high doses of moderate to vigorous physical activity, respectively. Physical activity is related to a 12% decline in the risk of cancers, when comparing people with the highest level with those with the lowest level of activity. Review findings also suggest that inactive older people are at greater risk of all-cause mortality, fractures, repeated falls, and functional restriction as compared to their active peers (19). Thus, physical inactivity imposes detrimental consequences on older adults' physical well-being.

Physical inactivity also has negative effects on psychological well-being. It has been observed that physical activity has a considerable effect in reducing the mental health related burden among older adults (16, 20, 21). According to the WHO, 2017, it is estimated that around 20% of older adults (60 years and above) suffer from a mental health or neurological disorder that

For peer review only - http://bmjopen.bmj.com/site/about/guidelines.xhtml

impacts their health. In addition, lack of physical activity has been linked to high levels of stress in older adults, whereas those older adults who are physically active report less stress (22). Similarly, physical activity has been identified as a cost-effective, efficient, and non-invasive way to reduce stress in older adults and to enhance their quality of life (23).

Evidence also supports a link between physical activity and reduced levels of depression in older adults. For instance, in their study including 1,123 Brazilian older adults (aged ≥ 60 years), Lage et al. (2021) identified that approximately 83.8% of participants demonstrated a decline in PA level, and that there is an inverse relationship between PA and depression. Participants who were engaged in daily moderate physical activity (β =−0.174; 95% CI = -0.026; -0.012) and moderate-to-vigorous physical activity (β = −0.183; 95% CI = −0.023; -0.011; (24) reported lower levels of depression than those who were not physically active. These findings corroborate a substantial body of prior evidence, including older adult participants and longitudinal explorations (25-27). Further Rethorst et al. (2017) also observed in women aged 18-74 years that substituting SB with vigorous PA was related to a reduction in depressive symptoms, but this was not the case for light or moderate intensity PA (28).

A significant association has also been uncovered between higher levels of anxiety and lower levels of self-reported PA in older adults (26). Reviews have indicated that moderate planned PA is strongly related to a decreased anxiety level (29). A nested case-control study with older community participants identified that those completing a high level of PA were less likely to suffer from cognitive impairment (OR = 0.58, 95% CI: 0.41–0.73; (30). Moreover, the combined evidence from different reviews signifies that PA might improve cognitive process as well as prevent cognitive impairment among older adults thus improving older people's quality of life through a range of mechanisms (31, 32). Therefore, encouraging older adults to be active and independent is a public health priority.

For peer review only - http://bmjopen.bmj.com/site/about/guidelines.xhtml

The UK guidelines on physical activity for adults suggest performing at least 150 minutes per week of moderate-intensity physical activity or 75 minutes per week of high-intensity physical activity (33). However, it has been reported that only 30% of older adults aged ≥ 75 and 55% of older individuals aged 55–74 meet the physical activity standards, as compared to 83% of adults in the age range of 18 to 24 years (34). This decline in PA among older adults has been attributed to numerous factors that include limited knowledge regarding PA benefits, lack of access to PA facilities, and lack of resources, time, and support (35-37). Literature highlights that housing is a major contributing factor to health, and better housing condition has been linked to improved general and psychological health (38, 39). In contrast, some advancements in the home setting, like the increased number of electronic media and devices, have reduced physical activity levels and enhanced sedentary behaviour, leading to detrimental consequences for public health (40). There has been strong evidence that the physical environment within the home space has a substantial impact on sedentary behaviour and physical activity in children (41). However, there is a lack of research that has investigated the physical setting within the home environment in the context of older adults' physical activity level and sedentary behaviour. Given that with increasing age, older adults spend large proportions of their time in their houses (42), it is important to optimize older adults' home settings to support healthy ageing, including engagement in PA and reductions in SB.

Moreover, in the current COVID-19 pandemic there is lack of access to community services and activities that has significantly affected older adults' life, with many finding it difficult to manage the repercussions of the pandemic, such as increased fear of being outside, and return to life as lived prior to the pandemic (43). Being home bound has led many older adults to spend their time watching television. A telephonic poll has showed that 73% of the older adults claimed that during pandemic they watched more television as compared to before the COVID-19 pandemic. Some have reported feeling guilt due to spending more time watching television

For peer review only - http://bmjopen.bmj.com/site/about/guidelines.xhtml

or playing videos and many older adults faced difficulties in maintaining their well-being, health and independence (43). The pandemic has therefore had a detrimental impact on their life in diverse ways by limiting their participation in different activities that are beneficial for their well-being (44). Older adults have experienced negative effects of staying at home, being isolated, feeling a lack of confidence and being scared about returning back to normal life (45). This has resulted in severe consequences for their physical and mental health and impaired their ability to independently perform their daily living activities. A survey showed that 42% of the older adults (>60 years) in the UK find it harder to walk up and down stairs and to walk short distances. Thirty six percent find it extremely challenging to take a shower or have a bath or wash whereas 43% of them were unable to prepare and cook food (44). Therefore, there is a need to enhance older adults' PA level within their home. This will encourage a healthy lifestyle among older people which in turn will increase their quality of life and foster their active participation in communities, reducing the personal and societal burden of physical inactivity and associated ill health (46). Moreover, little is known about older adults' perceptions and use of their home environment for reducing sedentary behaviour and increasing physical activity. Therefore, the current study aims to explore older adults' perceptions around optimizing their physical environment within their home space to support physical activity.

Methods and analysis:

Study design:

An exploratory qualitative research design using semi structured interviews and a purposive sampling approach will be employed in this study. In-depth interviews (IDIs) with older adults will be utilized as a data collection method for this qualitative research. The IDIs aim to explore perceptions of older adults towards optimizing their physical environment within their home

For peer review only - http://bmjopen.bmj.com/site/about/guidelines.xhtml

space to support physical activity. This study will be reported according to the guidelines for qualitative research provided in the consolidated checklist, Supplementary file 1.

Study setting and study participants:

The study will be conducted with older adults of different genders who will be selected based on the eligibility criteria (see below). In the first stage of participant recruitment different community organisations in Swansea, Bridgend and Neath Port Talbot will be approached to formally request approval to recruit via its contacts. Regional coordinators and sub-coordinators will act as gatekeepers as they will be approached for recruitment, to best ensure participating older adults reflect the typical socio-demographics of South Wales. Further, advertisement across the University using the intranet and work alongside the City and County of Swansea, Neath Port Talbot and Bridgend Borough Council will be done to recruit using their sport, play and community networks. Recruitment letters, posters/leaflets will be used as an enrolment technique.

The lead researcher will request approval from the project leads to make a visit to pitch for participants in the study. Interested older adults will be asked to express their interest via their respective area project co-ordinator. The researcher will contact interested older adults via email or phone. Older adults who agree to take part will receive participant information sheets, at which point a time for the researcher to meet them will be arranged that is mutually convenient and where the lead researcher will explain the study process and procedures if necessary. Providing the older adult is happy to proceed, they will be given a consent form to complete to carry out the interview.

Patient and public involvement:

There is no patient or public involvement in setting the research agenda.

For peer review only - http://bmjopen.bmj.com/site/about/guidelines.xhtml

In-depth interviews (IDIs) with older adults:

We will conduct IDIs with older adults to explore their perceptions towards optimizing their physical environment within their home space to support physical activity and reduce sedentary behaviour. IDI participants will be identified via the gatekeepers that will be approached by the researcher to seek permission to contact potential older adults via their organisation. All interested participants will be asked to further discuss the study and indicate their interest. The researcher will then organise a time for formal enrolment and induction to the study. All participants will provide written informed consent to participate in the study. The participants will be informed that the interview will be audio-recorded, that written notes might also be taken and they are not required to answer any questions they do not wish to. All IDI will be carried out in English and are anticipated to last around 30–40 minutes. Older adults will be informed that their data will remain confidential and there will be no identifying characteristics included on the verbatim-typed transcript. The main themes will consist of a general conversation about older adults' perceptions about optimizing their home environment to support PA and to explore the factors within older adults' home spaces that promote physical activity and well-being and reduce home based sedentary behaviours. We anticipate that 15–20 interviews will be carried out, however, interviews will be deemed complete based on the saturation of the data and no new themes emerge from the interviews. Therefore, data collection and analysis will occur simultaneously to determine the point of data saturation.

Eligibility criteria:

The following are the criteria for inclusion and exclusion of study participants:

Inclusion Criteria:

- Participants can be male or female living in their home
- All participants aged ≥ 65 years old

For peer review only - http://bmjopen.bmj.com/site/about/guidelines.xhtml

- Be able to communicate in English

Exclusion Criteria:

Participants will be excluded from the study if they:

- are not able to cooperate with the research team for the full duration of the project
- experience any physical/psychological issues that may put them at risk

Data collection procedure:

An in-depth interview guide has been developed for older adults. All older adults will have been provided with study information sheets and the study will be clearly explained to them. They will be told that participation is entirely voluntary, and they can withdraw at any point. All consenting participants will be interviewed. Each participant will be given a specific ID number which will be used throughout the study. A background survey will be designed to gather descriptive information about the older adults, that includes age, gender, education, and etc. This will be followed by the semi-structured interviews that will allow the researcher to obtain information about the participants' experiences of using and interacting with their home spaces to perform PA, their experiences of spending time in home and the impact on their physical activity. With regards to the older adults, we will also be specifically exploring activities they like to do. The interview will explore issues such as: How do they spend time at home? What kind of physical activity they perform at home? What are the ways of being physically active? They will also be asked about the number of days per week on which they were active for about 150-300 minutes, and the time they spend watching TV, sitting, and utilizing the internet for leisure time on a specific week or weekend day. They will be asked to discuss typical activities they carry out at home. They will be asked about their home environment, including topics such as how they selected their home and how it can be modified to enhance PA and lessen their sedentary behaviour at home. The impact of social isolation

For peer review only - http://bmjopen.bmj.com/site/about/guidelines.xhtml

and restricted activity will also be explored. All semi-structured interviews will be conducted either face-to-face or online via Zoom or WhatsApp depending on the older adult's feasibility. Based on the older adult's convenient day and time interviews will be scheduled. Interviews are anticipated to begin on mid of July 2022. The researcher will also keep a reflective diary. This will be utilised to record informal and incidental comments and observations, reflect on the recruitment and study process (including any feedback received from individuals who decline to participate) and to produce a reflective epilogue.

Patient and public involvement:

No patients will be involved.

Data analysis:

First, all collected data will be transcribed verbatim by the interviewer (lead researcher). Thematic analysis will be done and NVivo V.12 Plus software will be used to import, organise and explore data for analysis. To develop clarification and familiarity with the data two independent investigators will read the transcript several times. We will perform an iterative process that will guide us to categorize data and create new categories to generate emergent themes. The documented text will be separated into short units and labelled as a 'code' the reflects the main themes of interest in the study. Consequently, codes will be analysed and combined into similar categories, and these will be combined into subthemes and final themes. Two independent researchers will execute the coding, categories generation and thematic analysis in order to assure inter-rater reliability. To reduce researcher bias, disagreements between the two researchers will be settled through consensus sessions.

Ethics and dissemination:

Prior to participating in the study, older persons will be asked to submit written informed consent. Older adults who are not able to write down their names will be requested to give a

For peer review only - http://bmjopen.bmj.com/site/about/guidelines.xhtml

thumbprint to signify their willingness to participate. Ethical approval for this study has been obtained from the College of Engineering Research Ethics Committee (NM_31-03-22), Swansea University. The findings of the study will be disseminated to the scientific community in a peer reviewed journal article and to the study participants via workshops and seminar

Discussion:

The study findings will help us to understand and improve the geography of older adults' homes to enable them to be physically active and reduce their sedentary behaviour which in turn improves their health. It will help us to create awareness among older adults and widen the scope of the existing research to support the development of proper pathways in optimizing the home environment to support physical activity. In addition, research aims to inform social care policy that adopts a preventive healthcare model to maintain older adults' wellbeing by enabling them to be independent in their home surroundings.

review only

For peer review only - http://bmjopen.bmj.com/site/about/guidelines.xhtml

Contributor ship statement:

NAAM designed the protocol under the supervision of JH, GS and JM. JH & GS gave multiple critical feedback on overall draft. JM provided insights from her expertise in catering several research on older adults' population, which added more value in designing and revising protocol. All authors contributed to reviewing and editing the final draft.

Competing interests:
The authors declare that they have no competing interests.

Funding

This is funded by: Economic and Social Research Council (ES/P00069X/1)

Ethics approval

Ethical approval for this study has been obtained from the College of Engineering Research Ethics Committee (NM_31-03-22), Swansea University.

For peer review only

References:

1. Daskalopoulou C, Stubbs B, Kralj C, Koukounari A, Prince M, Prina AM. Physical activity and healthy ageing: A systematic review and meta-analysis of longitudinal cohort studies. Ageing research reviews. 2017;38:6-17.
2. World Health Organization (2017). Mental health of older adults. https://www.who.int/news-room/fact-sheets/detail/mental-health-of-older-adults.
3. McPhee JS, French DP, Jackson D, Nazroo J, Pendleton N, Degens H. Physical activity in older age: perspectives for healthy ageing and frailty. Biogerontology. 2016;17(3):567-80.
4. Zubritsky C, Abbott KM, Hirschman KB, Bowles KH, Foust JB, Naylor MD. Health-related quality of life: Expanding a conceptual framework to include older adults who receive long-term services and supports. The Gerontologist. 2013;53(2):205-10.
5. Hazra NC, Rudisill C, Gulliford MC. Determinants of health care costs in the senior elderly: age, comorbidity, impairment, or proximity to death? The European journal of health economics. 2018;19(6):831-42.
6. Ding D, Lawson KD, Kolbe-Alexander TL, Finkelstein EA, Katzmarzyk PT, Van Mechelen W, et al. The economic burden of physical inactivity: a global analysis of major non-communicable diseases. The Lancet. 2016;388(10051):1311-24.
7. Lee I-M, Shiroma EJ, Lobelo F, Puska P, Blair SN, Katzmarzyk PT, et al. Effect of physical inactivity on major non-communicable diseases worldwide: an analysis of burden of disease and life expectancy. The lancet. 2012;380(9838):219-29.
8. Bennett E, Clarke LH, Wolf SA, Dunlop W, Harden S, Liu Y, et al. Older adults' experiences of group-based physical activity: a qualitative study from the 'GOAL'randomized controlled trial. Psychology of Sport and Exercise. 2018;39:184-92.
9. Arem H, Matthews CE, Lee I-M. Physical Activity Is Key for Successful Aging—Reply: Even a Little Is Good. JAMA internal medicine. 2015;175(11):1863-.
10. Mummery K, Schofield G, Caperchione C. Physical activity: Physical activity dose-response effects on mental health status in older adults. Australian and New Zealand journal of public health. 2004;28(2):188-92.
11. Sala G, Jopp D, Gobet F, Ogawa M, Ishioka Y, Masui Y, et al. The impact of leisure activities on older adults' cognitive function, physical function, and mental health. PloS one. 2019;14(11):e0225006.
12. Izguttinov A. Community Readiness Assessment for the Dissemination of Evidence-based Physical Activity Programs for Older Adults in Astana, Kazakhstan 2019.
13. World Health Organization. Regional Office for Europe. (2012). Policies and priority interventions for healthy ageing. World Health Organization. Regional Office for Europe. https://apps.who.int/iris/handle/10665/349968.
14. World Health Organization. (2018). Global action plan on physical activity 2018– 2030: more active people for a healthier world. https://www.who.int/publications/i/item/9789241514187.
15. Schutzer KA, Graves BS. Barriers and motivations to exercise in older adults. Preventive medicine. 2004;39(5):1056-61.
16. Bherer L, Erickson KI, Liu-Ambrose T. A review of the effects of physical activity and exercise on cognitive and brain functions in older adults. Journal of aging research. 2013;2013.
17. Cadore EL, Rodriguez-Manas L, Sinclair A, Izquierdo M. Effects of different exercise interventions on risk of falls, gait ability, and balance in physically frail older adults: a systematic review. Rejuvenation research. 2013;16(2):105-14.
18. Lewis BA, Napolitano MA, Buman MP, Williams DM, Nigg CR. Future directions in physical activity intervention research: expanding our focus to sedentary behaviors, technology, and dissemination. Journal of behavioral medicine. 2017;40(1):112-26.

For peer review only - http://bmjopen.bmj.com/site/about/guidelines.xhtml

19. Cunningham C, O'Sullivan R, Caserotti P, Tully MA. Consequences of physical inactivity in older adults: A systematic review of reviews and meta-analyses. Scandinavian journal of medicine & science in sports. 2020;30(5):816-27.
20. Aguiñaga S, Ehlers DK, Salerno EA, Fanning J, Motl RW, McAuley E. Home-based physical activity program improves depression and anxiety in older adults. Journal of physical activity and health. 2018;15(9):692-6.
21. Mammen G, Faulkner G. Physical activity and the prevention of depression: a systematic review of prospective studies. American journal of preventive medicine. 2013;45(5):649-57.
22. McHugh JE, Lawlor BA. Exercise and social support are associated with psychological distress outcomes in a population of community-dwelling older adults. Journal of health psychology. 2012;17(6):833-44.
23. Churchill R, Riadi I, Kervin L, Teo K, Cosco T. Deciphering the role of physical activity in stress management during a global pandemic in older adult populations: a systematic review protocol. Systematic reviews. 2021;10(1):1-4.
24. Lage A, Carrapatoso S, Sampaio de Queiroz Neto E, Gomes S, Soares-Miranda L, Bohn L. Associations between depressive symptoms and physical activity intensity in an older adult population during COVID-19 lockdown. Frontiers in psychology. 2021;12:644106.
25. Fernandez-Alonso L, Muñoz-García D, La Touche R. The level of physical activity affects the health of older adults despite being active. Journal of exercise rehabilitation. 2016;12(3):194.
26. McKee G, Kearney PM, Kenny RA. The factors associated with self-reported physical activity in older adults living in the community. Age and ageing. 2015;44(4):586-92.
27. Yoshida Y, Iwasa H, Kumagai S, Suzuki T, Awata S, Yoshida H. Longitudinal association between habitual physical activity and depressive symptoms in older people. Psychiatry and clinical neurosciences. 2015;69(11):686-92.
28. Rethorst CD, Moncrieft AE, Gellman MD, Arredondo EM, Buelna C, Castañeda SF, et al. Isotemporal analysis of the association of objectively measured physical activity with depressive symptoms: results from Hispanic Community Health Study/Study of Latinos (HCHS/SOL). Journal of physical activity & health. 2017;14(9):733.
29. Sarris J, Moylan S, Camfield DA, Pase MP, Mischoulon D, Berk M, et al. Complementary medicine, exercise, meditation, diet, and lifestyle modification for anxiety disorders: a review of current evidence. Evidence-Based Complementary and Alternative Medicine. 2012;2012.
30. Laurin D, Verreault R, Lindsay J, MacPherson K, Rockwood K. Physical activity and risk of cognitive impairment and dementia in elderly persons. Archives of neurology. 2001;58(3):498-504.
31. Kohut M, McCann D, Russell D, Konopka D, Cunnick J, Franke W, et al. Aerobic exercise, but not flexibility/resistance exercise, reduces serum IL-18, CRP, and IL-6 independent of β-blockers, BMI, and psychosocial factors in older adults. Brain, behavior, and immunity. 2006;20(3):201-9.
32. Kadariya S, Gautam R, Aro AR. Physical activity, mental health, and wellbeing among older adults in South and Southeast Asia: a scoping review. BioMed research international. 2019;2019.
33. National Health Service (NHS). Health Check Programme, 2016. Best Practice Guidance. Department of Health. Available from. www.healthcheck.nhs.uk/document.php?o=1159, Accessed date: 25 August 2017.
34. British Heart Foundation, 2017. Physical Inactivity and Sedentary Behaviour Report.

Assessed 28/08/17.
35. Hartley SE, Yeowell G. Older adults' perceptions of adherence to community physical activity groups. Ageing & Society. 2015;35(8):1635-56.
36. Victor CR. Understanding physical activity in the daily lives of Bangladeshi and Pakistani elders in Great Britain. International Scholarly Research Notices. 2014;2014.
37. Lindsay Smith G, Banting L, Eime R, O'Sullivan G, Van Uffelen JG. The association between social support and physical activity in older adults: a systematic review. International Journal of Behavioral Nutrition and Physical Activity. 2017;14(1):1-21.

For peer review only

For peer review only - http://bmjopen.bmj.com/site/about/guidelines.xhtml

38. Thomson H, Thomas S, Sellstrom E, Petticrew M. The health impacts of housing improvement: a systematic review of intervention studies from 1887 to 2007. American journal of public health. 2009;99(S3):S681-S92.
39. Shaw M. Housing and public health. Annual review of public health. 2004;25:397.
40. Owen N, Healy GN, Matthews CE, Dunstan DW. Too much sitting: the population-health science of sedentary behavior. Exercise and sport sciences reviews. 2010;38(3):105.
41. Maitland C, Foster S, Stratton G, Braham R, Rosenberg M. Capturing the geography of children's active and sedentary behaviours at home: The HomeSPACE measurement tool. Children's Geographies. 2019;17(3):291-308.
42. Benzinger P, Iwarsson S, Kroog A, Beische D, Lindemann U, Klenk J, et al. The association between the home environment and physical activity in community-dwelling older adults. Aging clinical and experimental research. 2014;26(4):377-85.
43. Beaufort (2021), Older People in Wales Survey, data collected on behalf of the Older People's Commissioner for Wales, July 2021, 500 respondents aged 60+ living in Wales.
44. The Older People's Commissioner for Wales, 2021.State of the Nation 2021.

https://www.olderpeoplewales.com/en/reviews/sotn.aspx#:~:text=State%20of%20the%20Nation%3A%202021&text=The%20report%20found%20that%20whilst,the%20older%20population%20in%20Wales.
45. Beaufort. Older People in Wales Survey, data collected on behalf of the Older People's Commissioner for Wales, July 2021, 500 respondents aged 60+ living in Wales. 2021.
46. Welsh Assembly Government. (2008). The strategy for older people in Wales 2008- 2013. https://webarchive.nationalarchives.gov.uk/ukgwa/20080616133435/http:/new.wa.

For peer review only

For peer review only - http://bmjopen.bmj.com/site/about/guidelines.xhtml

Project Title:
Optimizing older adults home spaces to enhance their physical activity level: An exploratory qualitative study protocol

Demographic Information

1.Gender	
2. Age	
3.Your highest education level	1. No formal education<br>2. Less than primary school<br>3. Primary school completed<br>4. Secondary/High school completed<br>5. College/University completed<br>6. Post graduate degree
4. Your occupation	1. Government employee<br>2. Non-government employee<br>3. Self-employed<br>4. Non-paid<br>5. Retired<br>6. Unemployed (able to work)<br>7. Unemployed (unable to work)<br>8. Other, specify:
5. How many people live in your house now (**not** counting yourself)	____________ people<br>[insert number]
6. Number of families in the house:	1. One (i.e., husband and children)<br>2. Two or more (e.g., in-laws, brother-in-law and his family, other relatives like aunts, uncles)
7. What is your nationality?	1. Pakistani<br>2. Indian<br>3. Chinese<br>4. Bangladeshi<br>5. African<br>6. White<br>7. Other___________________________________
8. What is your current marital status?	1. Married<br>2. Living as couple (living together, but not legally married)<br>3. Divorced or separated<br>4. Single, never married<br>5. Widowed<br>6. Other (Specify):<br>___________________________________
9. Did you have any significant medical history? (check all that apply)	1. Heart attack<br>2. Heart surgery or cardiac angiogram or insertion of stents<br>3. Angina<br>4. Pacemaker or implantable cardiac defibrillator<br>5. Rhythm disturbance (irregular or fast heart rate)<br>6. Heart valve disease

For peer review only

	7. Heart failure<br>8. Heart transplantation<br>9. Congenital heart disease<br>10.High blood pressure<br>11. Diabetes<br>12. Renal disease<br>13. Asthma or any other lung condition<br>14. Any musculoskeletal (joint or muscle) problems that may limit your physical activity<br>15. Other _______________
10.Have you taken any prescription medications	1. Yes<br>2. No<br><br>If yes, please list the medications you have been taking:<br><br>_________________________

For peer review only

Indoors vs Outdoors

Some people describe themselves are being 'outdoorsy' or "indoorsy' people. How would you describe yourself? *Prompt: Would you say you are more indoorsy or outdoorsy? Do you like being active like walking in your spare time? Or doing things like watching TV, being on the computer or reading?*

Why do you say that? What makes you choose to do something active like walking in the garden vs watching TV ?

1. Thinking about your home. What are your favorite features of the house and garden ? What do you most like and why?
2. What do you most dislike and why?
3. What kind of activities do you perform at home?
4. Can you tell me the number of days in a usual week when you were physically active for at least 150-300 minutes or 2.5 hour to 5 hours?
5. What are your ways of being physically active at home?
6. What obstacles stand in the way of physical activity in your home setting?
7. Do you have access to mobile technology like laptops, smart phone, handheld electronic game etc?
8. Where do you usually use these at home? Where do you usually access the internet at home?
9. Can you tell me the time spent watching TV, playing video and computer games, and using the internet for leisure, on a typical week and weekend day?
10. Do you have any rules around media use we haven't already spoken about? Tell me about these. Prompt: When? Where? How long? Do these apply to the whole family?
11. Why did you choose this home for your family/yourself to live in? What things did you consider?
12. Why is your home organized in the way that it is at present?
13. Have you changed anything about your home over the past 5 years (e.g., technical equipment or device and the reason of these changes in home?
14. If you could change anything about your home, what would it be? Why would you do this?
15. What if you could redesign your house to do things like rearrange the rooms or make areas bigger or smaller? What would you do?
16. What if you had to make changes to the house and garden to make it more active? What would you do?
17. What if you had to make changes to the house & garden to get away from screens and spend less time on the couch? What would you do?
18. Do you have any physical activity equipment (treadmill, exercise bike, weights/resistance band) within your home?
19. Do you have exercise programming app (smartphone exercise apps, exergames, work out videos, virtual personal trainer, virtual workout groups)
20. Are there any parks nearby your home and would you prefer to go there?

For peer review only

21. What do you think that pandemic has restricted your physical activity level within your home surroundings? (Prompt: Is there any change in daily routine)
22. What do you think that currently your home physical environment (example space within home) promotes your health and well-being? (Prompt why? What are the factors?)
23. What are your suggestions and recommendations for older people in terms of optimizing their home space to be more physically active?

For peer review only

Additional file 1: COREQ 32-ITEM CHECKLIST

No. Item	Guide questions/description	Reported on Page #
Domain 1: Research team and reflexivity		
1. Inter viewer/facilitator	Which author/s conducted the interview?	Page # 11 (Data Collection Procedure)
2. Credentials	What were the researcher's credentials?	Page # 11(Data Collection Procedure)
3. Occupation	What was their occupation at the time of the study?	Page # 11 (Data Collection Procedure)
4. Gender	Was the researcher male or female?	Page # 9 (Data Collection Procedure)
5. Experience and training	What experience or training did the researcher have?	Page # 11 (Data Collection Procedure)
6. Relationship with participants established	Was a relationship established prior to study commencement?	Page # 11 (Data Collection Procedure)
7. Participant knowledge of the interviewer	What did the participants know about the researcher?	Page # 11 (Data Collection Procedure)
8. Interviewer characteristics	What characteristics were reported about the inter viewer/facilitator?	Page # 11 (Data Collection Procedure)
Domain 2: study design		
9. Methodological orientation and Theory	What methodological orientation was stated to underpin the study?	NA
10. Sampling	How were participants selected?	Page # 9 (Data Collection Methods and study participants)
11. Method of approach	How were participants approached?	Page # 9 (Data Collection Procedure)
12. Sample size	How many participants were in the study?	Page # Protocol (Result)
13. Non-participation	How many people refused to participate or dropped out? Reasons?	Page # Protocol (Result)
14. Setting of data collection	Where was the data collected?	Page # 11 (Data Collection Procedure)
15. Presence of non-participants	Was anyone else present besides the participants and researchers?	Page # 11 (Data Collection Procedure)
16. Description of sample	What are the important characteristics of the sample?	Page # Protocol (Result)
17. Interview guide	Were questions, prompts, guides provided by the authors?	In-Depth Interview Guide for interviewing students
18. Repeat interviews	Were repeat interviews carried out?	No
19. Audio/visual recording	Did the research use audio or visual recording to collect the data?	Page # 11 (Data Collection Procedure)
20. Field notes	Were field notes made during and/or	Page # 9 (Data Collection Procedure)

For peer review only

	after the interview?	
21. Duration	What was the duration of the interviews	Page # 11 (Data Collection Procedure)
22. Data saturation	Was data saturation discussed?	Page # Protocol (Result)
23. Transcripts returned	Were transcripts returned to participants for comment and/or correction?	No
Domain 3: analysis and findings		
24. Number of data coders	How many data coders coded the data?	Page # 12 (Data Analysis)
25. Description of the coding tree	Did authors provide a description of the coding tree?	NA
26. Derivation of themes	Were themes identified in advance or derived from the data?	Page # Protocol (Result)
27. Software	What software, if applicable, was used to manage the data?	Page # 12 (Data Analysis)
28. Participant checking	Did participants provide feedback on the findings?	No
29. Quotations presented	Were participant quotations presented to illustrate the themes/findings? Was each quotation identified?	Page # Protocol (Result)
30. Data and findings consistent	Was there consistency between the data presented and the findings?	Page # Protocol (Result)
31. Clarity of major themes	Were major themes clearly presented in the findings?	Page # Protocol (Result)
32. Clarity of minor themes	Is there a description of diverse cases or discussion of minor themes?	Page # Protocol (Result)

For peer review only

Optimizing older adults home spaces to enhance their physical activity level: An exploratory qualitative study protocol

Journal:	*BMJ Open*
Manuscript ID	bmjopen-2022-066940.R1
Article Type:	Protocol
Date Submitted by the Author:	30-Nov-2022
Complete List of Authors:	Meghani, Naureen; Swansea University College of Engineering - Bay Campus; Swansea University College of Engineering,<br>Hudson, Joanne; Swansea University College of Engineering - Bay Campus, Applied Sports Technology, Exercise and Medicine (A-STEM) Research Centre<br>Stratton, Gareth; Swansea University College of Engineering - Bay Campus, College of Engineering<br>Mullins, Jane; Swansea University School of Engineering
**Primary Subject Heading**:	Public health
Secondary Subject Heading:	Public health, Qualitative research, Mental health
Keywords:	QUALITATIVE RESEARCH, MENTAL HEALTH, Old age psychiatry < PSYCHIATRY

SCHOLARONE™
Manuscripts

TITLE PAGE

Study Title: Optimizing older adults home spaces to enhance their physical activity level: An exploratory qualitative study protocol

Naureen Akber Ali Meghani[1], Joanne Hudson[2], Gareth Stratton[3], Jane Mullins[4]

Details of the Authors:

[1]Naureen Akber Ali Meghani, PhD Student, Applied Sports Technology, Exercise and Medicine (A-STEM) Research Centre, Swansea University, Bay Campus, Swansea, United Kingdom, 2132644@ swansea.ac.uk . +07762330193

[2]Joanne Hudson, Professor of Exercise and Sport Psychology, Applied Sports Technology, Exercise and Medicine (A-STEM) Research Centre, Swansea University, Bay Campus, Swansea, United Kingdom, joanne.hudson@swansea.ac.uk 01792 513162

[3] Gareth Stratton, Chair in Paediatric Exercise Science, College of Engineering, Swansea University, Bay Campus, Swansea, United Kingdom, g.stratton@swansea.ac.uk 01792 516544

[4] Jane Mullins, Researcher, College of Engineering, Swansea University, Bay Campus, Swansea, United Kingdom, j.mullins@swansea.ac.uk 07954690170

Corresponding Author Detail:
Naureen Akber Ali Meghani
PhD Student,
Swansea University,
Bay Campus (SA1 8EP), Swansea, United Kingdom,
Cell Phone: +07762330193
Email: 2132644@swansea.ac.uk .

Keywords: Qualitative study, older adults, physical activity level, home spaces, exploratory study

Word Count: 3928

For peer review only

Abstract

Introduction:

Housing is a major contributing factor to health, and better housing condition has been linked to improved general and psychological health. There has also been strong evidence that the physical environment within the home setting substantially impacts sedentary behaviour and physical activity in children. However, there is a lack of research that has investigated the physical environment within the home setting in the context of older adults' physical activity levels and sedentary behaviour. Given that with increasing age, older adults spend large proportions of their time in their homes it is important to optimize older adults' home settings to support healthy ageing. Therefore, the current study aims to explore older adults' perceptions around optimizing their physical environment within their home space to support physical activity and subsequently facilitate healthy ageing.

Methods and analysis:

In this formative research, a qualitative exploratory research design using in-depth interviews (IDIs) and a purposive sampling approach will be employed. In-depth interviews will be conducted to collect data from study participants. Older adults from diverse community organizations in Swansea, Bridgend, and Neath Port Talbot will formally request approval to recruit via its contacts for this formative research. The study data will be analyzed thematically using NVivo V.12 Plus software.

Ethics and dissemination:

Ethical approval for this study has been obtained from the College of Engineering Research Ethics Committee (NM_31-03-22), Swansea University. The findings of the study will be disseminated to the scientific community and to the study participants. The results will enable

For peer review only

us to explore the perceptions and attitudes of older adults towards physical activity within their home environment.

Key words: Qualitative study, older adults, physical activity level, home spaces, exploratory study

Strengths and limitations of this study

- A well-established qualitative reflexive thematic analysis approach is used to understand in depth older adults' perception towards their physical activity within the home space.
- A social-ecological framework (Stokols,1992) that is based on physical aspects of the home will be used in the analysis of interviews.
- All semi-structured interviews will be conducted either face-to-face or online via Zoom or WhatsApp call depending on the older adult's feasibility. The call feature may restrict the researcher's ability to observe non-verbal cues during interviews.

For peer review only - http://bmjopen.bmj.com/site/about/guidelines.xhtml

Background:

Old age is defined as the most advanced stage of the biological cycle that is characterised by the manifestation of physical degradation and a general weakening of the organism. It is often linked with the state of inactivity and, subsequently, of need, as well as an excessive fragility from a health perspective (1). However, the extension of life expectancy due to scientific advancement means that it is essential to view old age from a wider perspective, taking into account that it is not only a phase in which mental and physical impairments might occur but also as an invaluable moment that can take on unique meaning (2). In a similar vein, older adults face transitions and change common to this phase of life, such as children leaving the home, retirement, the passing of friends and loved ones, sexuality and bodily changes, and the occurrence of illnesses (3). When it comes to ageing, the experience of space, especially the home space, becomes very important. Therefore, it is possible to plan and construct this space so that people will occupy it optimally and their home space will reflect and reinforce their identity (4). A qualitative analysis of old age, time, and the home space revealed that irrespective of loneliness, older adults expressed an emotional attachment with their living spaces, its role in connecting the memory of the past and life in general, reflecting a counter narrative to the stereotypical view of old age as characterised mainly by vulnerability (5).

Older adults appear to have a greater sense of meaning in life than younger people. Moreover, a study also showed that human relationships were noted as an important factor for strength and meaning in life among older adults with good cognitive functioning. Furthermore, engaging in new activities that provided meaning in life after retirement was more likely among high cognitive functioning older adults. The study also added that feeling worthless, along with poor health and physical functioning were significant contributors to a sense of meaninglessness of life among older people (6). A Korean study conducted on 198 older adults revealed strong relationships between meaning in life and health, depression and problematic

For peer review only - http://bmjopen.bmj.com/site/about/guidelines.xhtml

life events (7). Hence, health is an important factor for meaning in life and consequently for healthy ageing.

Similarly, generativity (such as feeling useful and needed) (8) can be considered as one indicator of a healthy ageing process (9). A study including older adults from diverse cultural backgrounds (German, Czech, Hong Kong Chinese and Cameroonian) showed a positive impact of generative concern on meaning in life, which was partially mediated by generative goals, after controlling for education level, competence, cognitive function and relationship (10). The perceptions of older adults surrounding key aspects of generativity (such as feeling useful and needed) may also be related to later-life health trajectories. In older Finnish birth cohorts, not feeling needed by others was associated with a higher chance of death and institutionalisation (over a 5 to 10 year period; 11). Similar results were found in rural-living French (60 years and above) and Japanese (65 years and above) older adults. Those with low perceptions of social usefulness experienced increases impairment in activities of daily living (ADLs) or died throughout follow-ups of 4 to 6 years (12-14). In sum, higher perceptions of generativity are linked to older persons' longer lifespans and better physical performance over time (8). Therefore, it is important to improve physical functioning and general health and well-being among older adults. Evidence suggests that physical activity (PA) is a modifiable behaviour linked to health, improved physical functional status, and longevity and promoting a physically active lifestyle has become a recognised public health objective (15, 16).

Physical activity (PA) is a key factor of healthy ageing; however, many older adults are not sufficiently physically active and have sedentary lifestyles which leads to detrimental health consequences, and even places financial and social constraints on themselves and society (17). This is a global concern, with longevity increasing around the world, it is estimated that proportion of older adults in the global population is expected to nearly double from roughly 12% to 22% between 2015 and 2050, translating to an increase from 900 million to 2 billion

For peer review only - http://bmjopen.bmj.com/site/about/guidelines.xhtml

people over the age of 60 (18). However, it is important to note that the aforementioned increase in life expectancy may not necessarily imply an increase in healthy life expectancy, as older people can face a variety of health issues (19). These include cognitive function decline, reduced functional movement, impaired mental health and chronic conditions such as diabetes, high blood pressure, and cardiovascular disease (19). These health issues can have an impact on an older person's quality of life (20), and increase healthcare costs and societal burden (e.g., medical care expenses; (21). According to estimates, physical inactivity contributes to the worldwide burden of noncommunicable diseases (NCDs), with health-care systems costs attributed to inactivity estimated at INT$53.8 billion globally, and further costs linked with INT$13.7 billion in productivity losses (22, 23). Reducing the mortality rates attributable to NCD calls for considerable action and equitable access to effective and good quality preventative and curative care, including behaviour modification such as increasing PA (24). In that regard, engagement in activity (as part of leisure, sport, work, and transportation) plays a significant role in successful ageing (25-27). Thus, developing strategies that are tailored to meet the needs of different subgroups (e.g., older adults) and that account for cultural contextual influences on behaviours related to, and attitudes towards, healthy ageing is paramount. Doing so will enable us to identify strategies to combat the effects of noncommunicable diseases, improve and preserve functional capacity, and promote physical and mental health among older persons to support independence and improve quality of life (27, 28). Therefore, there is an urgent need to increase older adults' PA levels and decrease their sedentary behaviour.

The World Health Organization has placed a particular emphasis on the promotion of PA to support healthy ageing (29). The Global Action Plan on Physical Activity 2018-2030 identifies the strategic goal of increasing PA opportunities to engage inactive populations such as older adults (30). Of concern, studies have shown that some older adults perceive

For peer review only - http://bmjopen.bmj.com/site/about/guidelines.xhtml

physical activity to be only beneficial to the younger population (31); however, there is compelling evidence that the requirement for physical activity does not end in later life (32). Doing so has numerous benefits, including a positive influence on the ability to perform the daily living activities (33, 34), and findings from longitudinal studies demonstrate that engaging in regular physical activity can improve cognitive ability among older adults (32). Findings from systematic review and meta-analysis showed that physically active older adults exhibited much lower arterial stiffness as compared to their sedentary peers (standardized mean difference: -1.017 ± 0.340, 95% CI: -1.684 to -0.350, $p = 0.003$). Further, there is a drastic decline in the risk of cardiovascular disease mortality from 25% to 40% that accompanies low and high doses of moderate to vigorous physical activity, respectively. Physical activity is related to a 12% decline in the risk of cancers, when comparing people with the highest level with those with the lowest level of activity. Review findings also suggest that inactive older people are at greater risk of all-cause mortality, fractures, repeated falls, and functional restriction as compared to their active peers (35). Thus, physical inactivity imposes detrimental consequences on older adults' physical well-being.

Physical inactivity also has negative effects on psychological well-being. It has been observed that physical activity has a considerable effect in reducing the mental health related burden among older adults (32, 36, 37). According to the WHO, 2017, it is estimated that around 20% of older adults (60 years and above) suffer from a mental health or neurological disorder that impacts their health. In addition, lack of physical activity has been linked to high levels of stress in older adults, whereas those older adults who are physically active report less stress (38). Similarly, physical activity has been identified as a cost-effective, efficient, and non-invasive way to reduce stress in older adults and to enhance their quality of life (39).

Evidence also supports a link between physical activity and reduced levels of depression in older adults. For instance, in their study including 1,123 Brazilian older adults (aged $\geq$ 60

For peer review only - http://bmjopen.bmj.com/site/about/guidelines.xhtml

years), Lage et al. (2021) identified that approximately 83.8% of participants demonstrated a decline in PA level, and that there is an inverse relationship between PA and depression. Participants who were engaged in daily moderate physical activity (β =−0.174; 95% CI = -0.026; -0.012) and moderate-to-vigorous physical activity (β = −0.183; 95% CI = −0.023; -0.011; (40) reported lower levels of depression than those who were not physically active. These findings corroborate a substantial body of prior evidence, including older adult participants and longitudinal explorations (41-43). Further Rethorst et al. (2017) also observed in women aged 18-74 years that substituting SB with vigorous PA was related to a reduction in depressive symptoms, but this was not the case for light or moderate intensity PA (44).

A significant association has also been uncovered between higher levels of anxiety and lower levels of self-reported PA in older adults (42). Reviews have indicated that moderate planned PA is strongly related to a decreased anxiety level (45). A nested case-control study with older community participants identified that those completing a high level of PA were less likely to suffer from cognitive impairment (OR = 0.58, 95% CI: 0.41–0.73; (46). Moreover, the combined evidence from different reviews signifies that PA might improve cognitive process as well as prevent cognitive impairment among older adults thus improving older people's quality of life through a range of mechanisms (47, 48). Therefore, encouraging older adults to be active and independent is a public health priority.

The UK guidelines on physical activity for adults suggest performing at least 150 minutes per week of moderate-intensity physical activity or 75 minutes per week of high-intensity physical activity (49). However, it has been reported that only 30% of older adults aged ≥ 75 and 55% of older individuals aged 55–74 meet the physical activity standards, as compared to 83% of adults in the age range of 18 to 24 years (50). This decline in PA among older adults has been attributed to numerous factors that include limited knowledge regarding PA benefits, lack of access to PA facilities, and lack of resources, time, and support (51-53). Literature highlights

For peer review only - http://bmjopen.bmj.com/site/about/guidelines.xhtml

that housing is a major contributing factor to health, and better housing condition has been linked to improved general and psychological health (54, 55). In contrast, some advancements in the home setting, like the increased number of electronic media and devices, have reduced physical activity levels and enhanced sedentary behaviour, leading to detrimental consequences for public health (56). There has been strong evidence that the physical environment within the home space has a substantial impact on sedentary behaviour and physical activity in children (57). However, there is a lack of research that has investigated the physical setting within the home environment in the context of older adults' physical activity level and sedentary behaviour. Given that with increasing age, older adults spend large proportions of their time in their houses (58), it is important to optimize older adults' home settings to support healthy ageing, including engagement in PA and reductions in SB.

Moreover, in the current COVID-19 pandemic there is lack of access to community services and activities that has significantly affected older adults' life, with many finding it difficult to manage the repercussions of the pandemic, such as increased fear of being outside, and return to life as lived prior to the pandemic (59). Being home bound has led many older adults to spend their time watching television. A telephonic poll has showed that 73% of the older adults claimed that during pandemic they watched more television as compared to before the COVID-19 pandemic. Some have reported feeling guilt due to spending more time watching television or playing videos and many older adults faced difficulties in maintaining their well-being, health and independence (59). The pandemic has therefore had a detrimental impact on their life in diverse ways by limiting their participation in different activities that are beneficial for their well-being (60). Older adults have experienced negative effects of staying at home, being isolated, feeling a lack of confidence and being scared about returning back to normal life (61). This has resulted in severe consequences for their physical and mental health and impaired their ability to independently perform their daily living activities. A survey showed that 42%

For peer review only - http://bmjopen.bmj.com/site/about/guidelines.xhtml

of the older adults (>60 years) in the UK find it harder to walk up and down stairs and to walk short distances. Thirty six percent find it extremely challenging to take a shower or have a bath or wash whereas 43% of them were unable to prepare and cook food (60). Therefore, there is a need to enhance older adults' PA level within their home. This will encourage a healthy lifestyle among older people which in turn will increase their quality of life and foster their active participation in communities, reducing the personal and societal burden of physical inactivity and associated ill health (62). Moreover, little is known about older adults' perceptions and use of their home environment for reducing sedentary behaviour and increasing physical activity. Therefore, the current study aims to explore older adults' perceptions around optimizing their physical environment within their home space to support physical activity.

Methods and analysis:

Study design:

An exploratory qualitative research design using semi structured interviews and a purposive sampling approach will be employed in this study from 1st August 2022 to 31st January 2023. In-depth interviews (IDIs) with older adults will be utilized as a data collection method for this qualitative research. The IDIs aim to explore perceptions of older adults towards optimizing their physical environment within their home space to support physical activity. This study will be reported according to the guidelines for qualitative research provided in the consolidated checklist, Supplementary file 1.

Study setting and study participants:

The study will be conducted with older adults of different genders who will be selected based on the eligibility criteria (see below). In the first stage of participant recruitment different community organisations in Swansea, Bridgend and Neath Port Talbot will be approached to

For peer review only - http://bmjopen.bmj.com/site/about/guidelines.xhtml

formally request approval to recruit via its contacts. Regional coordinators and sub-coordinators will act as gatekeepers as they will be approached for recruitment, to best ensure participating older adults reflect the typical socio-demographics of South Wales. Further, advertisement across the University using the intranet and work alongside the City and County of Swansea, Neath Port Talbot and Bridgend Borough Council will be done to recruit using their sport, play and community networks. Recruitment letters, posters/leaflets will be used as an enrolment technique.

The lead researcher will request approval from the project leads to make a visit to pitch for participants in the study. Interested older adults will be asked to express their interest via their respective area project co-ordinator. The researcher will contact interested older adults via email or phone. Older adults who agree to take part will receive participant information sheets, at which point a time for the researcher to meet them will be arranged that is mutually convenient and where the lead researcher will explain the study process and procedures if necessary. Providing the older adult is happy to proceed, they will be given a consent form to complete to carry out the interview.

Patient and public involvement:

There is no patient or public involvement in setting the research agenda.

In-depth interviews (IDIs) with older adults:

We will conduct IDIs with older adults to explore their perceptions towards optimizing their physical environment within their home space to support physical activity and reduce sedentary behaviour. IDI participants will be identified via the gatekeepers that will be approached by the researcher to seek permission to contact potential older adults via their organisation. All interested participants will be asked to further discuss the study and indicate their interest. The researcher will then organise a time for formal enrolment and induction to the study. All

For peer review only - http://bmjopen.bmj.com/site/about/guidelines.xhtml

participants will provide written informed consent to participate in the study. The participants will be informed that the interview will be audio-recorded, that written notes might also be taken and they are not required to answer any questions they do not wish to. All IDI will be carried out in English and are anticipated to last around 30–40 minutes. Older adults will be informed that their data will remain confidential and there will be no identifying characteristics included on the verbatim-typed transcript. The main themes will consist of a general conversation about older adults' perceptions about optimizing their home environment to support PA and to explore the factors within older adults' home spaces that promote physical activity and well-being and reduce home based sedentary behaviours. We anticipate that 15–20 interviews will be carried out, however, interviews will be deemed complete based on the saturation of the data and no new themes emerge from the interviews. Therefore, data collection and analysis will occur simultaneously to determine the point of data saturation.

Eligibility criteria:

The following are the criteria for inclusion and exclusion of study participants:

Inclusion Criteria:

- Participants can be male or female living in their home
- All participants aged ≥ 65 years old
- Be able to communicate in English

Exclusion Criteria:

Participants will be excluded from the study if they:

- are not able to cooperate with the research team for the full duration of the project
- experience any physical/psychological issues that may put them at risk

For peer review only - http://bmjopen.bmj.com/site/about/guidelines.xhtml

Data collection procedure:

An in-depth interview guide has been developed for older adults. All older adults will have been provided with study information sheets and the study will be clearly explained to them. They will be told that participation is entirely voluntary, and they can withdraw at any point. All consenting participants will be interviewed. Each participant will be given a specific ID number which will be used throughout the study. A background survey will be designed to gather descriptive information about the older adults, that includes age, gender, education, and etc. This will be followed by the semi-structured interviews that will allow the researcher to obtain information about the participants' experiences of using and interacting with their home spaces to perform PA, their experiences of spending time in home and the impact on their physical activity. With regards to the older adults, we will also be specifically exploring activities they like to do. The interview will explore issues such as: How do they spend time at home? What kind of physical activity they perform at home? What are the ways of being physically active? They will also be asked about the number of days per week on which they were active for about 150-300 minutes, and the time they spend watching TV, sitting, and utilizing the internet for leisure time on a specific week or weekend day. They will be asked to discuss typical activities they carry out at home. They will be asked about their home environment, including topics such as how they selected their home and how it can be modified to enhance PA and lessen their sedentary behaviour at home. The impact of social isolation and restricted activity will also be explored. All semi-structured interviews will be conducted either face-to-face or online via Zoom or WhatsApp depending on the older adult's feasibility. Based on the older adult's convenient day and time interviews will be scheduled. Interviews are anticipated to begin on mid of July 2022. The researcher will also keep a reflective diary. This will be utilised to record informal and incidental comments and observations, reflect on

For peer review only - http://bmjopen.bmj.com/site/about/guidelines.xhtml

For peer review only

the recruitment and study process (including any feedback received from individuals who decline to participate) and to produce a reflective epilogue.

Data analysis:

First, all collected data will be transcribed verbatim by the interviewer (lead researcher). Reflexive thematic analysis will be done and NVivo V.12 Plus software will be used to import, organise and explore data for analysis. To develop clarification and familiarity with the data two independent investigators will read the transcript several times. We will perform an iterative process that will guide us to categorize data and create new categories to generate emergent themes. The documented text will be separated into short units and labelled as a 'code' the reflects the main themes of interest in the study. Consequently, codes will be analysed and combined into similar categories, and these will be combined into subthemes and final themes based on the socio-ecological framework. The socio-ecological approach emphasises that an individual is not separate from the environment in which he or she spends their time, and the physical environment has an influence on the individual's behaviour, particularly PA (63, 64). Two independent researchers will execute the coding, categories generation and reflexive thematic analysis. To acknowledge researcher bias, disagreements between the two analysts will be settled through consensus sessions, with the second author acting as a critical friend, therefore challenging each other's interpretations.

Ethics and dissemination:

Prior to participating in the study, older persons will be asked to submit written informed consent. Older adults who are not able to write down their names will be requested to give a thumbprint to signify their willingness to participate. Ethical approval for this study has been obtained from the College of Engineering Research Ethics Committee (NM_31-03-22),

For peer review only - http://bmjopen.bmj.com/site/about/guidelines.xhtml

Swansea University. The findings of the study will be disseminated to the scientific community in a peer reviewed journal article and to the study participants via workshops and seminar

Discussion:

The physical environment is a modifiable factor, thus, optimising the physical setting for PA is crucial because individual change within the home environment can be an easily accessible way to drive significant changes without a facilitative infrastructure. The physical environment of the household is particularly important to older adults because it often determines opportunities for PA. The socio-ecological approach emphasises that an individual is not separate from the environment in which s/he spends their time, and the physical environment has an influence on the individual's behaviour, particularly PA (64). Moreover, the study findings will help us to understand and improve the geography of older adults' homes to enable them to be physically active and reduce their sedentary behaviour which in turn improves their health. It will help us to create awareness among older adults and widen the scope of the existing research to support the development of proper pathways in optimizing the home environment to support physical activity. Moreover, the results of this study can guide the designing and planning of specialised interventions aimed at encouraging older adults' physical activity within their home space. Furthermore, the findings of this study must take into account knowledge about what motivates older people to alter their behaviour as well as how to maintain a positive change in behaviour within their home environment to perform PA. Additionally, involving older adults should be a key aspect of any intervention to enhance the physical environment for physical activity, as older adults could offer invaluable insights into local need and could work together with the practitioners to increase the potential success of interventions. In addition, research aims to inform social care policy that adopts a preventive healthcare model to maintain older adults' wellbeing by enabling them to be independent in their home surroundings. This will encourage researchers, designers, developers, healthcare

For peer review only - http://bmjopen.bmj.com/site/about/guidelines.xhtml

experts, and policymakers to work together to identify and provide suitable and supportive housing environments and home spaces for an ageing population.

For peer review only - http://bmjopen.bmj.com/site/about/guidelines.xhtml

Contributor ship statement:

NAAM designed the protocol under the supervision of JH, GS and JM. JH & GS gave multiple critical feedback on overall draft. JM provided insights from her expertise in catering several research on older adults' population, which added more value in designing and revising protocol. All authors contributed to reviewing and editing the final draft.

Competing interests:

The authors declare that they have no competing interests.

Funding

This is funded by: Economic and Social Research Council (ES/P00069X/1)

Ethics approval

Ethical approval for this study has been obtained from the College of Engineering Research Ethics Committee (NM_31-03-22), Swansea University.

For peer review only

References:

1. 2018. Il Devoto-Oli. Vocabolario della lingua italiana [The Devoto-Oli. Vocabulary of the Italian language]. Milano: Le Monnier.
2. Monterosa L. Le intermittenze della vita [The intermittences of life]. In R. Corsa, L. Fattori, & G. Vandi (Eds.), Vecchiaia e psicoanalisi [Old age and psychoanalysis] Roma: Alpes.2020.
3. De Masi F. Psicoterapia in tarda età [Pychotherapy in late age]. In R. Corsa, L. Fattori, & G. Vandi (Eds.), Vecchiaia e psicoanalisi [Old age and psychoanalysis] Roma: Alpes.2020.
4. Eiguer A. L'inconscient de la maison [The unconscious of the house]. . Paris: Dunod.2004.
5. Lacatena M, Sommantico M. Old Age, Time, and the Space of the Home: A Qualitative Research Study During the Covid-19 Pandemic. Mediterranean Journal of Clinical Psychology. 2022;10(2).
6. Takkinen S, Ruoppila I. Meaning in life in three samples of elderly persons with high cognitive functioning. The International Journal of Aging and Human Development. 2001;53(1):51-73.
7. Chang SO, Patricia MB. Meaning in life among the elderly. Journal of Korean Academy of Nursing. 2000;30(2):259-71.
8. Gruenewald TL, Liao DH, Seeman TE. Contributing to others, contributing to oneself: Perceptions of generativity and health in later life. Journals of Gerontology Series B: Psychological Sciences and Social Sciences. 2012;67(6):660-5.
9. Schoklitsch A, Baumann U. Measuring generativity in older adults: The development of new scales. GeroPsych: The Journal of Gerontopsychology and Geriatric Psychiatry. 2011;24(1):31.
10. Hofer J, Busch H, Au A, Poláčková Šolcová I, Tavel P, Tsien Wong T. For the benefit of others: generativity and meaning in life in the elderly in four cultures. Psychology and aging. 2014;29(4):764.
11. Pitkala K, Laakkonen M-L, Strandberg TE, Tilvis RS. Positive life orientation as a predictor of 10-year outcome in an aged population. Journal of clinical epidemiology. 2004;57(4):409-14.
12. Grand A, Grosclaude P, Bocquet H, Pous J, Albarede J. Predictive value of life events, psychosocial factors and self-rated health on disability in an elderly rural French population. Social Science & Medicine. 1988;27(12):1337-42.
13. Grand A, Grosclaude P, Bocquet H, Pous J, Albaredez J. Disability, psychosocial factors and mortality among the elderly in a rural French population. Journal of clinical epidemiology. 1990;43(8):773-82.
14. Okamoto K, Tanaka Y. Subjective usefulness and 6-year mortality risks among elderly persons in Japan. The Journals of Gerontology Series B: Psychological Sciences and Social Sciences. 2004;59(5):P246-P9.
15. Haskell WL, Lee I-M, Pate RR, Powell KE, Blair SN, Franklin BA, et al. Physical activity and public health: updated recommendation for adults from the American College of Sports Medicine and the American Heart Association. Circulation. 2007;116(9):1081.
16. US Department of Health and Human Services, Office of Disease Prevention and Health Promotion. Physical activity guidelines for Americans. http://www.health.gov/paguidelines. Accessed March 25, 2009 [
17. Daskalopoulou C, Stubbs B, Kralj C, Koukounari A, Prince M, Prina AM. Physical activity and healthy ageing: A systematic review and meta-analysis of longitudinal cohort studies. Ageing research reviews. 2017;38:6-17.
18. World Health Organization (2017). Mental health of older adults. https://www.who.int/news-room/fact-sheets/detail/mental-health-of-older-adults.
19. McPhee JS, French DP, Jackson D, Nazroo J, Pendleton N, Degens H. Physical activity in older age: perspectives for healthy ageing and frailty. Biogerontology. 2016;17(3):567-80.
20. Zubritsky C, Abbott KM, Hirschman KB, Bowles KH, Foust JB, Naylor MD. Health-related quality of life: Expanding a conceptual framework to include older adults who receive long-term services and supports. The Gerontologist. 2013;53(2):205-10.

For peer review only - http://bmjopen.bmj.com/site/about/guidelines.xhtml

21. Hazra NC, Rudisill C, Gulliford MC. Determinants of health care costs in the senior elderly: age, comorbidity, impairment, or proximity to death? The European journal of health economics. 2018;19(6):831-42.
22. Ding D, Lawson KD, Kolbe-Alexander TL, Finkelstein EA, Katzmarzyk PT, Van Mechelen W, et al. The economic burden of physical inactivity: a global analysis of major non-communicable diseases. The Lancet. 2016;388(10051):1311-24.
23. Lee I-M, Shiroma EJ, Lobelo F, Puska P, Blair SN, Katzmarzyk PT, et al. Effect of physical inactivity on major non-communicable diseases worldwide: an analysis of burden of disease and life expectancy. The lancet. 2012;380(9838):219-29.
24. Bennett E, Clarke LH, Wolf SA, Dunlop W, Harden S, Liu Y, et al. Older adults' experiences of group-based physical activity: a qualitative study from the 'GOAL'randomized controlled trial. Psychology of Sport and Exercise. 2018;39:184-92.
25. Arem H, Matthews CE, Lee I-M. Physical Activity Is Key for Successful Aging—Reply: Even a Little Is Good. JAMA internal medicine. 2015;175(11):1863-.
26. Mummery K, Schofield G, Caperchione C. Physical activity: Physical activity dose-response effects on mental health status in older adults. Australian and New Zealand journal of public health. 2004;28(2):188-92.
27. Sala G, Jopp D, Gobet F, Ogawa M, Ishioka Y, Masui Y, et al. The impact of leisure activities on older adults' cognitive function, physical function, and mental health. PloS one. 2019;14(11):e0225006.
28. Izguttinov A. Community Readiness Assessment for the Dissemination of Evidence-based Physical Activity Programs for Older Adults in Astana, Kazakhstan 2019.
29. World Health Organization. Regional Office for Europe. (2012). Policies and priority interventions for healthy ageing. World Health Organization. Regional Office for Europe. https://apps.who.int/iris/handle/10665/349968.
30. World Health Organization. (2018). Global action plan on physical activity 2018– 2030: more active people for a healthier world. https://www.who.int/publications/i/item/9789241514187.
31. Schutzer KA, Graves BS. Barriers and motivations to exercise in older adults. Preventive medicine. 2004;39(5):1056-61.
32. Bherer L, Erickson KI, Liu-Ambrose T. A review of the effects of physical activity and exercise on cognitive and brain functions in older adults. Journal of aging research. 2013;2013.
33. Cadore EL, Rodriguez-Manas L, Sinclair A, Izquierdo M. Effects of different exercise interventions on risk of falls, gait ability, and balance in physically frail older adults: a systematic review. Rejuvenation research. 2013;16(2):105-14.
34. Lewis BA, Napolitano MA, Buman MP, Williams DM, Nigg CR. Future directions in physical activity intervention research: expanding our focus to sedentary behaviors, technology, and dissemination. Journal of behavioral medicine. 2017;40(1):112-26.
35. Cunningham C, O'Sullivan R, Caserotti P, Tully MA. Consequences of physical inactivity in older adults: A systematic review of reviews and meta-analyses. Scandinavian journal of medicine & science in sports. 2020;30(5):816-27.
36. Aguiñaga S, Ehlers DK, Salerno EA, Fanning J, Motl RW, McAuley E. Home-based physical activity program improves depression and anxiety in older adults. Journal of physical activity and health. 2018;15(9):692-6.
37. Mammen G, Faulkner G. Physical activity and the prevention of depression: a systematic review of prospective studies. American journal of preventive medicine. 2013;45(5):649-57.
38. McHugh JE, Lawlor BA. Exercise and social support are associated with psychological distress outcomes in a population of community-dwelling older adults. Journal of health psychology. 2012;17(6):833-44.
39. Churchill R, Riadi I, Kervin L, Teo K, Cosco T. Deciphering the role of physical activity in stress management during a global pandemic in older adult populations: a systematic review protocol. Systematic reviews. 2021;10(1):1-4.

For peer review only - http://bmjopen.bmj.com/site/about/guidelines.xhtml

40. Lage A, Carrapatoso S, Sampaio de Queiroz Neto E, Gomes S, Soares-Miranda L, Bohn L. Associations between depressive symptoms and physical activity intensity in an older adult population during COVID-19 lockdown. Frontiers in psychology. 2021;12:644106.
41. Fernandez-Alonso L, Muñoz-García D, La Touche R. The level of physical activity affects the health of older adults despite being active. Journal of exercise rehabilitation. 2016;12(3):194.
42. McKee G, Kearney PM, Kenny RA. The factors associated with self-reported physical activity in older adults living in the community. Age and ageing. 2015;44(4):586-92.
43. Yoshida Y, Iwasa H, Kumagai S, Suzuki T, Awata S, Yoshida H. Longitudinal association between habitual physical activity and depressive symptoms in older people. Psychiatry and clinical neurosciences. 2015;69(11):686-92.
44. Rethorst CD, Moncrieft AE, Gellman MD, Arredondo EM, Buelna C, Castañeda SF, et al. Isotemporal analysis of the association of objectively measured physical activity with depressive symptoms: results from Hispanic Community Health Study/Study of Latinos (HCHS/SOL). Journal of physical activity & health. 2017;14(9):733.
45. Sarris J, Moylan S, Camfield DA, Pase MP, Mischoulon D, Berk M, et al. Complementary medicine, exercise, meditation, diet, and lifestyle modification for anxiety disorders: a review of current evidence. Evidence-Based Complementary and Alternative Medicine. 2012;2012.
46. Laurin D, Verreault R, Lindsay J, MacPherson K, Rockwood K. Physical activity and risk of cognitive impairment and dementia in elderly persons. Archives of neurology. 2001;58(3):498-504.
47. Kohut M, McCann D, Russell D, Konopka D, Cunnick J, Franke W, et al. Aerobic exercise, but not flexibility/resistance exercise, reduces serum IL-18, CRP, and IL-6 independent of β-blockers, BMI, and psychosocial factors in older adults. Brain, behavior, and immunity. 2006;20(3):201-9.
48. Kadariya S, Gautam R, Aro AR. Physical activity, mental health, and wellbeing among older adults in South and Southeast Asia: a scoping review. BioMed research international. 2019;2019.
49. National Health Service (NHS). Health Check Programme, 2016. Best Practice Guidance. Department of Health. Available from. www.healthcheck.nhs.uk/document.php?o=1159, Accessed date: 25 August 2017.
50. British Heart Foundation, 2017. Physical Inactivity and Sedentary Behaviour Report.

Assessed 28/08/17.
51. Hartley SE, Yeowell G. Older adults' perceptions of adherence to community physical activity groups. Ageing & Society. 2015;35(8):1635-56.
52. Victor CR. Understanding physical activity in the daily lives of Bangladeshi and Pakistani elders in Great Britain. International Scholarly Research Notices. 2014;2014.
53. Lindsay Smith G, Banting L, Eime R, O'Sullivan G, Van Uffelen JG. The association between social support and physical activity in older adults: a systematic review. International Journal of Behavioral Nutrition and Physical Activity. 2017;14(1):1-21.
54. Thomson H, Thomas S, Sellstrom E, Petticrew M. The health impacts of housing improvement: a systematic review of intervention studies from 1887 to 2007. American journal of public health. 2009;99(S3):S681-S92.
55. Shaw M. Housing and public health. Annual review of public health. 2004;25:397.
56. Owen N, Healy GN, Matthews CE, Dunstan DW. Too much sitting: the population-health science of sedentary behavior. Exercise and sport sciences reviews. 2010;38(3):105.
57. Maitland C, Foster S, Stratton G, Braham R, Rosenberg M. Capturing the geography of children's active and sedentary behaviours at home: The HomeSPACE measurement tool. Children's Geographies. 2019;17(3):291-308.
58. Benzinger P, Iwarsson S, Kroog A, Beische D, Lindemann U, Klenk J, et al. The association between the home environment and physical activity in community-dwelling older adults. Aging clinical and experimental research. 2014;26(4):377-85.
59. Beaufort (2021), Older People in Wales Survey, data collected on behalf of the Older People's Commissioner for Wales, July 2021, 500 respondents aged 60+ living in Wales.
60. The Older People's Commissioner for Wales, 2021.State of the Nation 2021.

For peer review only - http://bmjopen.bmj.com/site/about/guidelines.xhtml

https://www.olderpeoplewales.com/en/reviews/sotn.aspx#:~:text=State%20of%20the%20Nation%3A%202021&text=The%20report%20found%20that%20whilst,the%20older%20population%20in%20Wales.
61. Beaufort. Older People in Wales Survey, data collected on behalf of the Older People's Commissioner for Wales, July 2021, 500 respondents aged 60+ living in Wales. 2021.
62. Welsh Assembly Government. (2008). The strategy for older people in Wales 2008- 2013. https://webarchive.nationalarchives.gov.uk/ukgwa/20080616133435/http:/new.wa.
63. Bakhtari F, Ahmad B, Aminisani N, Gilani N, Allahverdipour H. Psychological, social, and environmental predictors of physical activity among older adults: The socio-ecological approach using structural equation modeling analysis. Baltic Journal of Health and Physical Activity. 2019;11(2):12.
64. Stokols D. Establishing and maintaining healthy environments: Toward a social ecology of health promotion. American psychologist. 1992;47(1):6.

For peer review only

Project Title:
Optimizing older adults home spaces to enhance their physical activity level: An exploratory qualitative study protocol

Demographic Information

1.GENDER	
2. AGE	
3.Your highest education level	1. No formal education<br>2. Less than primary school<br>3. Primary school completed<br>4. Secondary/High school completed<br>5. College/University completed<br>6. Post graduate degree
4. Your occupation	1. Government employee<br>2. Non-government employee<br>3. Self-employed<br>4. Non-paid<br>5. Retired<br>6. Unemployed (able to work)<br>7. Unemployed (unable to work)<br>8. Other, specify:
5. How many people live in your house now (**not** counting yourself)	____________ people<br>[insert number]
6. Number of families in the house:	1. One (i.e., husband and children)<br>2. Two or more (e.g., in-laws, brother-in-law and his family, other relatives like aunts, uncles)
7. What is your nationality?	1. Pakistani<br>2. Indian<br>3. Chinese<br>4. Bangladeshi<br>5. African<br>6. White<br>7. Other___________________________________
8. What is your current marital status?	1. Married<br>2. Living as couple (living together, but not legally married)<br>3. Divorced or separated<br>4. Single, never married<br>5. Widowed<br>6. Other (Specify):<br>____
9. Did you have any significant medical history? (check all that apply)	1. Heart attack<br>2. Heart surgery or cardiac angiogram or insertion of stents<br>3. Angina<br>4. Pacemaker or implantable cardiac defibrillator<br>5. Rhythm disturbance (irregular or fast heart rate)<br>6. Heart valve disease

For peer review only

	7. Heart failure<br>8. Heart transplantation<br>9. Congenital heart disease<br>10.High blood pressure<br>11. Diabetes<br>12. Renal disease<br>13. Asthma or any other lung condition<br>14. Any musculoskeletal (joint or muscle) problems that may limit your physical activity<br>15. Other ________________
10.Have you taken any prescription medications	1. Yes<br>2. No<br><br>If yes, please list the medications you have been taking:<br><br>__________________________

For peer review only

Indoors vs Outdoors

Some people describe themselves are being 'outdoorsy' or “indoorsy’ people. How would you describe yourself? *Prompt: Would you say you are more indoorsy or outdoorsy? Do you like being active like walking in your spare time? Or doing things like watching TV, being on the computer or reading?*

Why do you say that? What makes you choose to do something active like walking in the garden vs watching TV ?

1. Thinking about your home. What are your favorite features of the house and garden ? What do you most like and why?
2. What do you most dislike and why?
3. What kind of activities do you perform at home?
4. Can you tell me the number of days in a usual week when you were physically active for at least 150-300 minutes or 2.5 hour to 5 hours?
5. What are your ways of being physically active at home?
6. What obstacles stand in the way of physical activity in your home setting?
7. Do you have access to mobile technology like laptops, smart phone, handheld electronic game etc?
8. Where do you usually use these at home? Where do you usually access the internet at home?
9. Can you tell me the time spent watching TV, playing video and computer games, and using the internet for leisure, on a typical week and weekend day?
10. Do you have any rules around media use we haven’t already spoken about? Tell me about these. Prompt: When? Where? How long? Do these apply to the whole family?
11. Why did you choose this home for your family/yourself to live in? What things did you consider?
12. Why is your home organized in the way that it is at present?
13. Have you changed anything about your home over the past 5 years (e.g., technical equipment or device and the reason of these changes in home?
14. If you could change anything about your home, what would it be? Why would you do this?
15. What if you could redesign your house to do things like rearrange the rooms or make areas bigger or smaller? What would you do?
16. What if you had to make changes to the house and garden to make it more active? What would you do?
17. What if you had to make changes to the house & garden to get away from screens and spend less time on the couch? What would you do?
18. Do you have any physical activity equipment (treadmill, exercise bike, weights/resistance band) within your home?
19. Do you have exercise programming app (smartphone exercise apps, exergames, work out videos, virtual personal trainer, virtual workout groups)
20. Are there any parks nearby your home and would you prefer to go there?

For peer review only

21. What do you think that pandemic has restricted your physical activity level within your home surroundings? (Prompt: Is there any change in daily routine)
22. What do you think that currently your home physical environment (example space within home) promotes your health and well-being? (Prompt why? What are the factors?)
23. What are your suggestions and recommendations for older people in terms of optimizing their home space to be more physically active?

For peer review only

Additional file 1: COREQ 32-ITEM CHECKLIST

No. Item	Guide questions/description	Reported on Page #
Domain 1: Research team and reflexivity		
1. Inter viewer/facilitator	Which author/s conducted the interview?	Page # 11 (Data Collection Procedure)
2. Credentials	What were the researcher's credentials?	Page # 11(Data Collection Procedure)
3. Occupation	What was their occupation at the time of the study?	Page # 11 (Data Collection Procedure)
4. Gender	Was the researcher male or female?	Page # 9 (Data Collection Procedure)
5. Experience and training	What experience or training did the researcher have?	Page # 11 (Data Collection Procedure)
6. Relationship with participants established	Was a relationship established prior to study commencement?	Page # 11 (Data Collection Procedure)
7. Participant knowledge of the interviewer	What did the participants know about the researcher?	Page # 11 (Data Collection Procedure)
8. Interviewer characteristics	What characteristics were reported about the inter viewer/facilitator?	Page # 11 (Data Collection Procedure)
Domain 2: study design		
9. Methodological orientation and Theory	What methodological orientation was stated to underpin the study?	NA
10. Sampling	How were participants selected?	Page # 9 (Data Collection Methods and study participants)
11. Method of approach	How were participants approached?	Page # 9 (Data Collection Procedure)
12. Sample size	How many participants were in the study?	Page # Protocol (Result)
13. Non-participation	How many people refused to participate or dropped out? Reasons?	Page # Protocol (Result)
14. Setting of data collection	Where was the data collected?	Page # 11 (Data Collection Procedure)
15. Presence of non-participants	Was anyone else present besides the participants and researchers?	Page # 11 (Data Collection Procedure)
16. Description of sample	What are the important characteristics of the sample?	Page # Protocol (Result)
17. Interview guide	Were questions, prompts, guides provided by the authors?	In-Depth Interview Guide for interviewing students
18. Repeat interviews	Were repeat interviews carried out?	No
19. Audio/visual recording	Did the research use audio or visual recording to collect the data?	Page # 11 (Data Collection Procedure)
20. Field notes	Were field notes made during and/or	Page # 9 (Data Collection Procedure)

For peer review only

	after the interview?	
21. Duration	What was the duration of the interviews	Page # 11 (Data Collection Procedure)
22. Data saturation	Was data saturation discussed?	Page # Protocol (Result)
23. Transcripts returned	Were transcripts returned to participants for comment and/or correction?	No
Domain 3: analysis and findings		
24. Number of data coders	How many data coders coded the data?	Page # 12 (Data Analysis)
25. Description of the coding tree	Did authors provide a description of the coding tree?	NA
26. Derivation of themes	Were themes identified in advance or derived from the data?	Page # Protocol (Result)
27. Software	What software, if applicable, was used to manage the data?	Page # 12 (Data Analysis)
28. Participant checking	Did participants provide feedback on the findings?	No
29. Quotations presented	Were participant quotations presented to illustrate the themes/findings? Was each quotation identified?	Page # Protocol (Result)
30. Data and findings consistent	Was there consistency between the data presented and the findings?	Page # Protocol (Result)
31. Clarity of major themes	Were major themes clearly presented in the findings?	Page # Protocol (Result)
32. Clarity of minor themes	Is there a description of diverse cases or discussion of minor themes?	Page # Protocol (Result)

For peer review only
